# Evaluation of Preloaded IOL Delivery System and Hydrophobic Acrylic Intraocular Lens in Cataract Surgery

**DOI:** 10.2174/1874364101812010094

**Published:** 2018-06-14

**Authors:** Banu Acar, Isil M. Torun, Suphi Acar

**Affiliations:** 1 Bati Goz Hospital, Istanbul, Turkey; 2 Igdir State Hospital, Ophthalmology Clinic, Merkez, Igdir, Turkey

**Keywords:** IOL, Cataract surgery, Visual acuity, Preloaded delivery system, Hydrophobic IOL, Contrast sensitivity

## Abstract

**Background:**

Advancements in cataract surgery have necessitated the availability of intraocular lens preloaded delivery systems that can safely, effectively and predictably deliver IOLs in the eye. Preloaded delivery systems simplify and reduce procedural variability during surgery preparation.

**Objective:**

The objective of this study was to evaluate clinical acceptability, delivery characteristics and clinical outcomes in patients implanted with new generation hydrophobic acrylic Intraocular Lens with Preloaded delivery system.

**Methods:**

This was a single centre retrospective study. Total 41 patients were enrolled in the study to get at least 38 patients for evaluation. All patients were assessed on day1 and 1, 3 and 6 months after surgery.

**Results:**

EYECRYL-SERT showed ‘excellent’ ease of insertion and handling in all 41(100%) patients. Corrected Distance Visual Acuity (CDVA) improved from 0.74±0.58 logMAR during screening to 0.03±0.04 logMAR 6 months after surgery. The Corrected Intermediate Visual Acuity (CIVA) and Corrected Near Visual Acuity (CNVA) were 0.10±0.04 and 0.01±0.02 logMAR post 6 months surgery, respectively. The refractive spherical equivalence was -1.94±2.51 D during screening, which improved significantly (p=0.0018) to -0.21±0.47 D post 6 months surgery. The low and high contrast sensitivity was 0.06±0.06 and -0.05±0.06 logMAR after 6 months surgery, respectively. The endothelial cell loss was 5.67%, 7.22% and 9.75% at 1, 3, and 6 months after surgery, respectively, as compared to screening. None of the subjects reported any adverse event during the study period.

**Conclusion:**

The IOL delivery system (EYECRYL_SERT) provided desired delivery characteristics during cataract surgery and was effective in improving clinical outcomes in cataract patients.

## 1 INTRODUCTION

A cataract is a clouding of the lens in the eye affecting the vision and is the most common cause of blindness [1, 2]. The prevalence of cataract is burgeoning with age and affects about 40% of adults with age ≥70 years and growing to 60% of adults having age above 75 years [3]. Cataract caused worldwide 33.4% of all blindness in 2010 and 18.4% Moderate to Severe Vision Impairment (MSVI) [4]. Cataract surgery is the only effective treatment available to restore or maintain a vision for cataract [5, 6].

With the development of surgical techniques and biomaterial science, cataract surgery with Intraocular Lens (IOL) implantation has become a standard procedure and offers great benefits for patients. However, Posterior Capsule Opacification (PCO) remains the most frequent long-term complication, decreasing the visual performance in 1 or 2 years after cataract surgery [7]. Currently, acrylic and silicone foldable IOLs are available and used in small-incision cataract surgery. Between these two, acrylic lenses lead to a lower incidence of PCO and a higher rate of IOL stability in the bag [8]. Even in the acrylic IOLs, hydrophobic IOL are associated with lesser PCO and glare problems than hydrophilic IOL. Further, hydrophobic material has better capsular biocompatibility than hydrophilic material. That is why acrylic hydrophobic IOLs are used widely nowadays [9, 10].

Although serious complications are uncommon in cataract surgery, manually loading of hydrophilic acrylic lenses increases the time of surgery and number of surgical errors [11]. The Preloaded IOL delivery systems simplify and reduce procedural variability during surgery preparation. The implantation of the lens using injector is possible through the smaller incision size which is impossible with forceps. The small incision size with this technique results in shorter duration of wound healing, faster recovery, and reduced risk of infection by the reduction of micro-organism access in the early postoperative period [12, 13]. The trends of rigid polymethyl methylacrylates are being declined and utilization of soft silicone or acrylic lenses which are foldable is increasing. The risk for bacterial entry into the eye is reduced because the foldable IOL makes no direct contact with the incision or the operative field [14]. The further advancement of injectable IOLs into preloaded IOL delivery system has many potential advantages. It avoids the loading error and surgical error, loading variability seen in non-preloaded systems and reduces surgical time. The delivery of IOL in the capsular bag prevents manipulation during surgery [15].

This retrospective study assessed the delivery characteristic and predictability in patients implanted with new generation preloaded folded Eyecryl SERT (Biotech Vision Care, Ahmedabad, India) delivery system for its easy insertion, handling and incision size, unfolding time in capsular bag after surgery, visual acuity, contrast sensitivity, and endothelial cell count.

## 2 MATERIALS AND METHODS

### 2.1 Study Design and Participants

This was an observational, retrospective study to evaluate clinical acceptability and delivery characteristics of new Preloaded IOL Delivery System and the clinical outcomes in patients implanted with new generation hydrophobic acrylic Intraocular Lens with Preloaded delivery system EYECRYL SERT. The study was conducted in accordance with ICH-GCP, ISO 14155, Medical Device Directives of Global Harmonization Task Force and European Union, and all the pertinent Confidential and Proprietary local regulations. The study was performed in accordance with the Declaration of Helsinki. The study protocol was approved by the Independent Ethics Committee (IEC) [Haydarpasa Numune Research and Training Hospital Ethical Committee, Uskudar / Istanbul, Turkey (Permit Number: HNEAH-KAEK 2015/249)].

Total 41 eyes of 41 patients who met the following inclusion criteria were included in the study: 1) Unilateral/Bilateral diagnosed cataract, 2) Patients who had undergone cataract surgery and implanted with the study device and 3) Patient who had attended all the regular follow-up examinations as per the routine schedule. Patients who had any of the following criteria were excluded from the study: 1) Standard exclusion criteria for cataract surgery, 2) Corneal astigmatism greater than 1 D and 3) Pre-existing retinal disease.

### 2.2 Study Procedure

EYECRYL-SERT, the new generation of hydrophobic acrylic Intraocular Lens with Preloaded delivery system, was implanted in the enrolled patients. Concomitant treatments were given to patients as a standard medication treatment after cataract surgery. All patients were assessed as per their follow-ups schedule on day1, 1 month, 3 months, and 6 months after surgery.

The investigational variables related to the delivery system included ease of insertion and handling and incision size required. The unfolding time of preloaded IOL was also calculated during surgery.

Data collected from preoperative assessments included: Uncorrected Distance Visual Acuity (UDVA) and Corrected Distance Visual Acuity (CDVA) tested with ETDRS charts at 4m, refractive status as Manifest Spherical Equivalent (MSE; value of the sphere plus one-half of the value of the cylinder) and optical biometry measurements. Biometry was performed with IOL Master (Carl Zeiss, Jena, Germany). Manual biometry was utilized in eyes in which IOL master could not be performed because of a dense cataract. The IOL power was calculated with SRK-T formula (A-constant: 118.5) in eyes with an Axial Length (AL) of 22 to 24 mm. Hoffer Q formula (pACD = 5.61) was used in eyes with a shorter AL, and Holladay 2 formula (ACD constant = 5.607) was used in the eyes with a longer AL (> 24 mm).

Postoperative data collected at regular one, three and six-month visits included UDVA and CDVA with ETDRS charts at 4m, Uncorrected Near Visual Acuity (UNVA) and Corrected Near Visual Acuity (CNVA) with the Jaeger card at 40cm, Uncorrected Intermediate Visual Acuity (UIVA) and Corrected Intermediate Visual Acuity (CIVA) with the Jaeger card at 80 cm. Refractive status was recorded as in preoperative assessment. Scotopic and photopic contrast sensitivity testing with ETDRS charts at 80 cm was also recorded. Additionally, the endothelial cell count was measured by Konan Cell Check.

### 2.3 Statistical Considerations

We had assumed that the previous course of therapy gave around 50% ease in using the device while the improved device will give around 76% satisfaction in using the device. Forty-one eyes of 41 patients were required to detect the difference between observed and expected responder rate of the test product with around 90% confidence interval and 5% significance level. Considering 10% dropout rate around 42 numbers of patients were required to enroll in the study. Detailed descriptive analyses of the study endpoints were performed after each follow-up time point. All calculations were based on available data with missing data excluded. Any unused or spurious data were noted as appropriate in the final report.

## 3 RESULTS

A total of 41 patients were enrolled in the study with a mean (± SD) age of 66.22(± 9.33) years. Out of 41 patients, 23 (56.10%) were male and 18 (43.90%) female, respectively. Majority of the patients *i.e.*, 30 (73.17%) had age > 60 years. Out of the 41 patients, 40 completed the study and one patient was terminated because the patient did not follow regular post-operative examination schedule.

### 3.1 Ease of Insertion and Handling, Incision Size and Unfolding Time

Ease of insertion and handling during surgery were classified as excellent, very good, good and need improvement. We observed ‘excellent’ ease of insertion and handling during surgery for EYECRYL-SERT in all the 41(100%) study patients. The median incision size required during the surgery was 2.2 mm. The minimum incision size was 2.2 mm and maximum incision size was 2.8 mm. The majority of patients 39 (95.12%) required incision size between 2.2 to 2.4 mm during surgery, while only 2 (4.88%) patients required >2.4 mm incision. The unfolding time noted was 2.83 (± 0.50) seconds with a minimum of 2 seconds and maximum of 4 seconds.

### 3.2 Visual Acuity

#### 3.2.1 Uncorrected Distance Visual Acuity

The uncorrected visual acuity results are presented in Table **1**. There was a statistically significant improvement in Uncorrected Distance Visual Acuity (UDVA) from 1 month to 3 months and 1 month to 6 months (Table **1**, p=0.0107, p ≤0.001). At 6 months, median DCNVA was 0.48 ±0.04 logMAR (Table **1**).

#### 3.2.2 Corrected Distance Visual Acuity

The corrected visual acuity results are presented in Table **2**. The statistically significant improvement (p< 0.0001) was noted for CDVA after 1 month, 3 months and 6 months of surgery as compared to screening period. At 6 months, median DCNVA was 0.01 ±0.02 logMAR (Table **2**).

### 3.3 Refraction

The mean refractive spherical equivalence was -1.94±2.51 D during screening phase, which improved significantly to -0.30±0.45 D (p=0.0022), -0.22±0.46 D (p=0.0016), and -0.21±0.47 D (p=0.0018) after 1 month, 3 months, and 6 months of surgery, respectively. Mean SE was stable across the 1, 3, and 6 month visits, and there were no statistically significant differences between the postoperative visits (Table **3**).

### 3.4 Leading Haptic Position

The leading haptic position was found in the bag and no manipulation/dialing was required during the surgery for all the patients. Average time to achieve satisfactory IOL position was ≤2 seconds.

### 3.5 Contrast Sensitivity

**Fig. (1):**
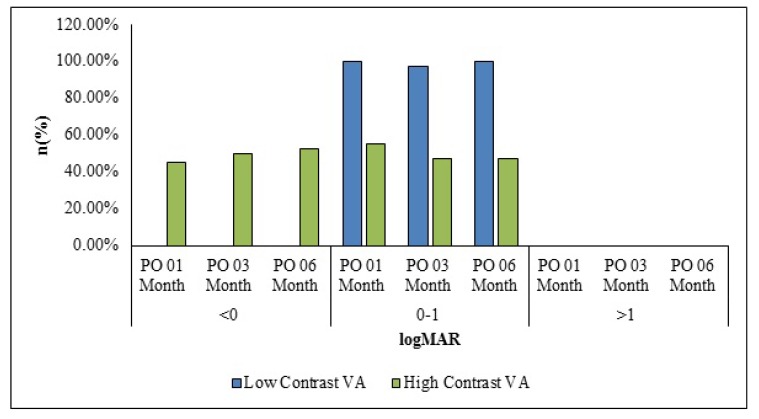
Contrast Sensitivity - FAS Population.

Low contrast sensitivity was 0.09±0.12 logMAR after 1 month of surgery which decreased to 0.06±0.07 logMAR every 3 and 6 months after surgery. The mean± SD value for high contrast sensitivity was -0.03±0.08 logMAR 1-month after surgery which decreased to -0.05±0.06 logMAR each 3 and 6 months after surgery. Forty (97.56%) patients had low contrast sensitivity in the range of 0.0 to 0.2 logMAR at 6 months after surgery (Fig. **1**). The high contrast sensitivity range was -0.1 to 0.1 logMAR in the 40 (97.56%) patients at 6 months after surgery.

### 3.6 Endothelial Cell Count

**Fig. (2):**
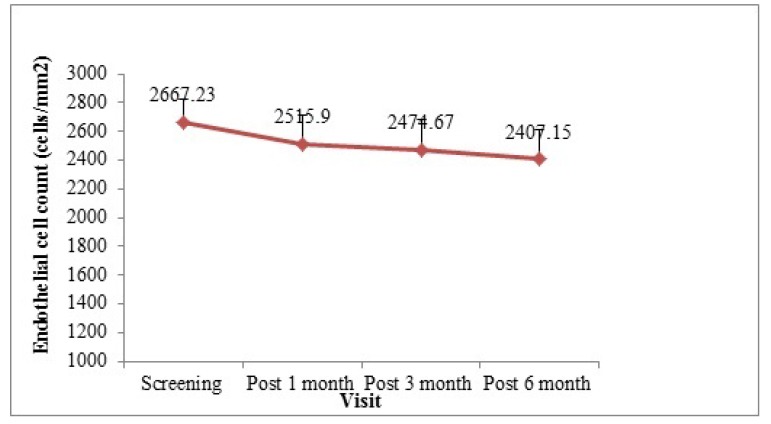
Endothelial Cell Count (cells/mm^2^) by Visit.

The endothelial cell count (mean±SD) was 2667.23±160.48 cells/mm^2^ during screening visit which decreased to 2515.90±199.65, 2474.67±205.19, and 2407.15±205.33 cells/mm^2^ after 1 month, 3 months, and 6 months of surgery, respectively (Fig. **2**). The difference from the screening visit was -156.89±144.36, -208.85±172.15, and -268.49±186.56 cells/mm^2^ after 1 month, 3 months, and 6 months of surgery, respectively (p<0.0001). The endothelial cell loss was -5.67%, -7.22%, and -9.75% 1 month, 3 months, and 6 months after surgery compared to the endothelial cell presented during the screening phase.

### 3.7 Keratometry

**Fig. (3):**
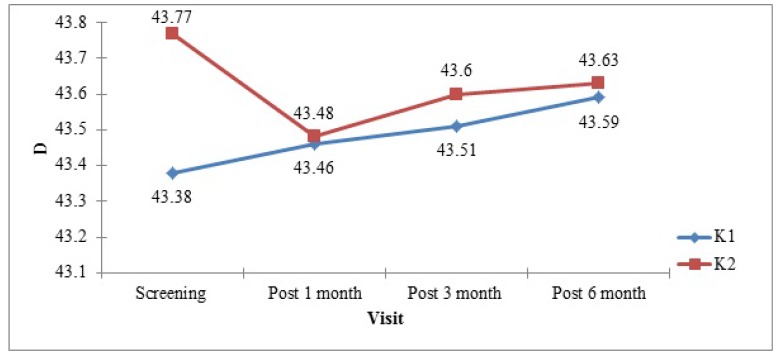
Keratometry Observation by Visit (FAS Population).

Additionally, keratometry was also performed at screening, after 1 month, 3 months and 6 months of surgery. The K1 value *(mean±SD)* was 43.38±1.68 D during screening period which was 43.46±1.36, 43.51±1.37, and 43.59±1.36 D after 1 month, 3 months, and 6 months of surgery respectively (Fig. **3**). The K2 value (mean±SD) was 43.77±1.82 D during screening period which at 1 month, 3 months, and 6 months after surgery was 43.48±1.61 D, 43.60±1.52 D, and 43.63±1.51 D, respectively. In all the subjects, K1 and K2 values were between 35-50 D.

No adverse events and adverse device effects reported during the study.

## 4 DISCUSSION

It has been reported that ocular surface bacteria contaminate the aqueous humor in 7-43% of cataract operations [16, 17]. Every contact of IOL and surgical fluid with surgical instruments increases the risk of bacterial contamination from surgical field which is not completely sterile. With a preloaded IOL system, we eliminate the loading IOL into the cartridge, thereby reduceing the number of contact IOL with surgical instruments, which helps to avoid any possible bacteria contamination which may possibly end up in endophthalmitis. In our study, we did not report any infection after surgery. We did not consider observing any infections following surgery because only using the preloaded system but using the preloaded IOL system can be considered as an additional protective approach for cataract surgery. We also used prophylactic methods including intracameral antibiotic injection and intraoperative disinfection uses against any bacterial contamination.

The ease of insertion and handling was found excellent in all the subjects in this study. In another study of hydrophobic acrylic lens, authors reported similar ease of handling with the preloaded system [18]. The preloaded system was attributed to a reduction in serial lens handling and preparation activities. The mean time of surgery (incision to closure time) was 7.6 ± 0.58 minutes. The IOL with lower unfolding time is required to reduce the risk and complications arising due to longer unfolding time. The IOL with longer unfolding time may lead to difficulty in the insertion of IOL from an injection system into capsular bag and even the possibilities of incomplete unfolding exist [5]. In this study, the unfolding time was 2.83 ± 0.50 seconds and none of the case-patients required IOL manipulation to achieve satisfactory IOL position. In a study of Preloaded AcrySof IQ SN60WS/AcrySert, only 38 of the 85 eyes (45%) studied achieved satisfactory IOL position without the need for additional manipulation. This variation may be attributed to characteristics of the delivery system and subjective difference. Because of increased manipulation of foldable IOLs within their injectors, the IOLs are more prone to damage [14].

We observed statistically significant improvement (p<0.0001) in CDVA after 1 month, 3 months, and 6 months of surgery compared with the screening value. None of the subjects had logMAR values more than 0.5 after 1 month, 3 months, and 6 months of surgery for CDVA, CIVA, and CINA. In a study conducted by Kretz *et al*. also, there was a significant improvement (p<0.001) in CDVA from 0.16 logMAR to 0.04 logMAR postoperatively [19]. In another study, Lee *et al*. compared the clinical outcomes of three different aspheric intraocular lenses (Tecnis^®^ Z9003, Acrysof^®^ IQ, and Adapt^®^ AO) and showed similar improvement in BCVA 6 months after surgery [20]. Moreover, the refractive spherical equivalence in our study was -1.94±2.51 D which increased significantly to -0.21±0.47 D after 6 months (p=0.0018) of surgery, proving the improvement in the vision. Significant improvement in SE was also reported by Kretz *et al*. in their study after implantation of trifocal IOL [21].

In this study, low and high contrast sensitivity was found to be within normal range after intraocular lens implantation (0.06 ±0.06 logMAR and -0.05±0.06 logMAR, respectively, after 6 months of surgery).

The endothelial cell loss was 5.67%, 7.22%, and 9.75% after 1 month, 3 months, and 6 months of surgery compared to endothelial cell at screening. It is known that phacoemulsification surgery decreases endothelial cell loss more than normal again process. Reduction rate of endothelial cell loss with aging was reported 0.3% and 1% annually in some studies [22, 23]. However, mean endothelial cell loss is reported to be 5.41% at 6 weeks post-operatively by George R. *et al.* 11% at 1 month postoperatively by Perjone JM *et al.* l and 17.92% with temporal Clear Corneal Incision (CCI) technique and 15.38% by superior Scleral Incision (SI) technique by Jangani SN [24-26]. All these values are similar or higher than our results. Walkow *et al.* have similar results and they reported that the mean overall central endothelial cell loss in all eyes was 8.5%, 12 months after surgery which is also compatible with our results [27]. In terms of endothelial cell loss, our results agree with other studies; however, long-term follow-up requires to better evaluate the effect on endothelial cell count.

None of the subjects in our study had any adverse event [24], during the study period, which is consistent with the other similar studies reported for preloaded IOL delivery system [11, 18].

## CONCLUSION

In summary, EYECRYL_SERT preloaded intraocular lens was associated with ease of insertion and handling and required lesser incision size, unfolding time and provided good distance, intermediate, and near VA post-surgery. The haptic position was present in the capsular bag during implantation, hence no manipulation was required during surgery. The spherical and cylindrical refraction was also improved after IOL implantation. In addition, none of the subjects reported any adverse event.

## Figures and Tables

**Table 1:** Summary of Uncorrected Visual Acuity by Visit (FAS Population).

**Parameter**	**Descriptive** **Statistics**	**EYECRYL-SERT (N=41)**		
		**PO 01** **Month**	**PO 03** **Month**	**PO 06** **Month**
Uncorrected Distance VA (Log MAR)	N	40	39	40
-	Mean (SD)	0.18 (0.11)	0.15 (0.07)	0.12 (0.08)
	Median	0.2	0.2	0.1
	Min, Max	0.0, 0.5	0.0, 0.2	0.0, 0.2
	p-value	-	0.0107	<.0001
Uncorrected Distance VA Ref Range [n (%)]	0-1	40 (97.56%)	39 (95.12%)	40 (97.56%)
-	>1	0 (0.00%)	0 (0.00%)	0 (0.00%)
Uncorrected Intermediate VA (Log MAR)	N	40	39	40
-	Mean (SD)	0.47 (0.08)	0.47 (0.04)	0.48 (0.04)
	Median	0.5	0.5	0.5
	Min, Max	0.3, 0.8	0.4, 0.5	0.4, 0.5
	p-value	-	0.7500	0.7500
Uncorrected Intermediate VA Ref Range [n (%)]	0-1	40 (97.56%)	39 (95.12%)	40 (97.56%)
-	>1	0 (0.00%)	0 (0.00%)	0 (0.00%)
Uncorrected Near VA (Log MAR)	N	40	39	40
-	Mean (SD)	0.35 (0.11)	0.38 (0.04)	0.38 (0.04)
	Median	0.4	0.4	0.4
	Min, Max	0.0, 0.6	0.3, 0.4	0.3, 0.4
	p-value	-	0.2031	0.2500
Uncorrected Near VA Ref Range [n (%)]	0-1	40 (97.56%)	39 (95.12%)	40 (97.56%)
-	>1	0 (0.00%)	0 (0.00%)	0 (0.00%)
**Abbreviations**: N=number of subjects in the specified treatment; n=number of subjects in the specified category; VA=visual acuity. Note 1: The given interval is inclusive of both values. Note 2: When screening visit data is not available we have considered 'PO 01 Month' visit data as baseline. Note 3: All parameters shows non-normality hence p-values obtained by using Wilcoxon signed-rank test.				

**Table 2:** Summary of Corrected Visual Acuity by Visit (FAS Population).

**Parameter**	**Descriptive** **Statistics**	**EYECRYL-SERT (N=41)**			
		**Screening**	**PO 01** **Month**	**PO 03** **Month**	**PO 06** **Month**
Corrected Distance VA (Log MAR)	N	40	40	39	40
-	Mean (SD)	0.74 (0.58)	0.05 (0.07)	0.04 (0.06)	0.03 (0.04)
	Median	0.5	0.0	0.0	0.0
	Min, Max	0.2, 2.6	0.0, 0.2	0.0, 0.2	0.0, 0.1
	p-value	-	<.0001	<.0001	<.0001
Corrected Distance VA Ref Range [n (%)]	0-1	35 (85.37%)	40 (97.56%)	39 (95.12%)	40 (97.56%)
-	>1	5 (12.20%)	0 (0.00%)	0 (0.00%)	0 (0.00%)
Corrected Intermediate VA (Log MAR)	N	-	40	39	40
-	Mean (SD)	-	0.11 (0.08)	0.10 (0.04)	0.10 (0.04)
	Median	-	0.1	0.1	0.1
	Min, Max	-	0.0, 0.5	0.0, 0.2	0.0, 0.2
	p-value	-	-	1.0000	1.0000
Corrected Intermediate VA Ref Range [n (%)]	0-1	-	40 (97.56%)	39 (95.12%)	40 (97.56%)
-	>1	-	0 (0.00%)	0 (0.00%)	0 (0.00%)
Corrected Near VA (Log MAR)	N	-	40	39	40
-	Mean (SD)	-	0.01 (0.06)	0.01 (0.02)	0.01 (0.02)
	Median	-	0.0	0.0	0.0
	Min, Max	-	0.0, 0.4	0.0, 0.1	0.0, 0.1
	p-value	-	-	1.0000	1.0000
Corrected Near VA Ref Range [n (%)]	0-1	-	40 (97.56%)	39 (95.12%)	40 (97.56%)
-	>1	-	0 (0.00%)	0 (0.00%)	0 (0.00%)
**Abbreviations**: N=number of subjects in the specified treatment; n=number of subjects in the specified category; VA=visual acuity. '-'=Not applicable. Note 1: The given interval is inclusive of both values. Note 2: When screening, visit data was not available. We considered 'PO 01 Month' visit data as baseline. Note 3: All the parameters show non-normality hence p-values obtained by using Wilcoxon signed-rank test.					

**Table 3:** Summary of Manifest Refraction by Visit (FAS Population).

-		**EYECRYL-SERT (N=41)**			
**Parameter**	**Descriptive** **Statistics**	**Screening**	**PO 01** **Month**	**PO 03** **Month**	**PO 06** **Month**
Sphere	N	30	40	39	40
	Mean (SD)	-1.35 (2.52)	0.01 (0.50)	0.10 (0.48)	0.10 (0.55)
	Median	-1.00	0.00	0.00	0.00
	Min, Max	-7.00, 6.00	-1.00, 1.00	-1.00, 1.00	-1.00, 1.50
	p-value	-	0.0123	0.0078	0.0090
Sphere Ref Range [n(%)]	< -0.5	19 (46.34%)	3 (7.32%)	2 (4.88%)	2 (4.88%)
	-0.5 - +0.5	7 (17.07%)	30 (73.17%)	30 (73.17%)	29 (70.73%)
	> +0.5	4 (9.76%)	7 (17.07%)	7 (17.07%)	9 (21.95%)
Cylinder	N	30	40	39	40
	Mean (SD)	-1.18 (1.22)	-0.62 (0.52)	-0.63 (0.55)	-0.62 (0.51)
	Median	-1.00	-0.50	-0.75	-0.50
	Min, Max	-5.50, 0.50	-2.00, 0.00	-2.00, 0.50	-2.00, 0.00
	p-value	-	0.0138	0.0174	0.0181
Cylinder Ref Range [n(%)]	< -0.5	19 (46.34%)	18 (43.90%)	20 (48.78%)	18 (43.90%)
	-0.5 - +0.5	11 (26.83%)	22 (53.66%)	19 (46.34%)	22 (53.66%)
	> +0.5	0 (0.00%)	0 (0.00%)	0 (0.00%)	0 (0.00%)
**Abbreviations**: N=number of subjects in the specified treatment; n=number of subjects in the specified category. Note 1: The given interval is inclusive of both values. Note 2: When screening, visit data was not available. We considered 'PO 01 Month' visit data as baseline. Note 3: p-value for parameter sphere obtained by using paired t-test and for cylinder by using Wilcoxon signed rank test.					
